# Exploring odontocete depredation rates in a pelagic longline fishery

**DOI:** 10.1371/journal.pone.0301072

**Published:** 2024-03-28

**Authors:** Eric Gilman, Milani Chaloupka, Aude Pacini, Eric Kingma

**Affiliations:** 1 Fisheries Research Group, United States of America; 2 Ecological Modelling Services and Marine Spatial Ecology Lab, University of Queensland, Brisbane, Australia; 3 University of Hawaii, Honolulu, HI, United States of America; 4 Hawaii Longline Association, Honolulu, HI, United States of America; Universidade Federal de Mato Grosso do Sul, BRAZIL

## Abstract

Several odontocete species depredate catch and bait from fishing gear, resulting in their bycatch and causing substantial economic costs. There are no known mitigation methods for odontocete depredation in pelagic longline fisheries that are effective, do not harm odontocetes and are commercially viable. Understanding odontocetes’ depredation strategies can contribute to mitigating this human-wildlife conflict. Using observer data from the Hawaii-based tuna longline fishery, this study summarized teleost and elasmobranch species-specific mean posterior odontocete depredation rates using a simple Bayesian binomial likelihood estimator with a Bayes-Laplace prior. Depredation rates of species with sufficient sample sizes ranged from a high of 1.2% (1.1 to 1.3 95% highest posterior density interval or HDI) for shortbill spearfish to a low of 0.002% (0.001 to 0.003 95% HDI) for blue shark. Depredation of catch is a rare event in this fishery, occurring in about 6% of sets. When depredation did occur, most frequently odontocetes depredated a small proportion of the catch, however, there was large variability in depredation rates between teleost species. For example, bigeye tuna was two times more likely to be depredated than yellowfin tuna (odds ratio = 2.03, 95% CI: 1.8–2.3, P<0.0001). For sets with depredation, 10% and 2% of sets had depredation of over half of the captured bigeye tuna and combined teleosts, respectively. All elasmobranch species had relatively low depredation rates, where only 7 of almost 0.5M captured elasmobranchs were depredated. Odontocetes selectively depredate a subset of the teleost species captured within sets, possibly based on net energy value, chemical, visual, acoustic and textural characteristics and body size, but not median length, which was found to be unrelated to depredation rate (Pearson’s r = 0.14, 95% CI: -0.26 to 0.50, p = 0.49). Study findings provide evidence to support the identification and innovation of effective and commercially viable methods to mitigate odontocete depredation and bycatch.

## 1. Introduction

Marine mammals occupy a range of trophic levels and some species have important roles in regulating the structure, function, dynamics and state of marine communities and ecosystems [1–6]. Large changes in their abundance can therefore cause profound community- and ecosystem-level consequences that are protracted or irreversible. Some marine mammals, including sirenians and most toothed whales, are extremely sensitive to anthropogenic mortality due to having relatively low rates of potential population growth and other intrinsic life history characteristics, small population sizes, and extrinsic environmental variables such as narrow habitat requirements for foraging and breeding [7–9].

Fisheries bycatch in both in-use and derelict (abandoned, lost and discarded) fishing gear is the largest threat to many populations of marine megafauna, including marine mammals, and is an obstacle to sustainable seafood production [9–12]. Of the range of anthropogenic hazards faced by marine mammals, bycatch affects the greatest number of species, contributed to the presumed extinction of the baiji (Yangtze River dolphin, *Lipotes vexillifer*), and has contributed to reducing the abundance of several populations and species of cetaceans and pinnipeds to critical levels [9, 13, 14]. Furthermore, marine mammal depredation (removal and damage of catch and bait from fishing gear) causes substantial direct and indirect socioeconomic costs [15, 16]. Depredation can also result in fishers deliberately injuring and killing marine mammals, and alters marine mammals’ foraging behavior, distribution, diet and demographics [7, 15–19].

Several species of odontocetes (Odontoceti, toothed whales), including the false killer whale (*Pseudorca crassidens*), short-finned pilot whale (*Globicephala macrorhynchus*) and killer whale (*Orcinus orca*), depredate catch and bait in pelagic longline fisheries, which can result in their bycatch by becoming hooked or entangled in line [20, 21]. A U.S. central north Pacific Ocean tuna longline fishery with vessels based primarily from Hawaii experiences odontocete depredation and bycatch primarily by false killer whales [22]. Under the U.S. Marine Mammal Protection Act, a Take Reduction Team was established in 2010 because the five-year running average of the estimated annual false killer whale mortalities and series injuries (M&SI) exceeded a threshold Potential Biological Removal (PBR) level. The team was tasked with reducing M&SI to below PBR within six months and to a level approaching zero, defined as <10% of PBR, within five years, and was further tasked with having no increase in M&SIs for a high seas false killer whale stock [23]. To date, existing measures recommended by the team and adopted by the US fisheries management authority (‘weak’ circle hooks, permanent static closed area, fleetwide bycatch cap that triggers an area closure, prescribed handling and release practices [23]) have not achieved <10% of PBR, and M&SIs on the high seas have increased [22]. More effective false killer whale depredation and bycatch mitigation methods are needed.

Unlike for some other threatened taxonomic groups exposed to bycatch in pelagic longline fisheries, there are no known odontocete methods across the tiers of a sequential bycatch mitigation hierarchy (avoid, minimize, remediate and offset) that are: (1) effective, (2) do not harm odontocetes, and (3) have acceptable costs to commercial viability (economic viability, practicality and crew safety) [15, 21, 24, 25]. There is low strength of evidence of the efficacy and commercial viability of bycatch and depredation mitigation approaches for odontocetes in pelagic longline gear. This includes mitigation approaches such as spatiotemporal fisheries management, including quasi-real time dynamic (fleet communication, move-on rules, habitat suitability modelling, real-time acoustic tracking) and static approaches [9, 15, 22, 24, 26, 27], input controls such as limits on soak and haul duration [24, 28], and output controls such as bycatch thresholds [29]. The same applies to gear technology approaches such as:

- Catch encasement to physically protect catch from depredation, and visual and acoustic camouflage of target catch such as by using bubble screens or knots [24, 30, 31];
- Mainline length limits, hookless sections of mainline, hookless sets, and set geometry [32];
- Active acoustic deterrents and decoys, including pingers, acoustic harassment devices, decoy vessels, broadcasts of decoy fishing vessel acoustic cues, broadcasts of killer whale and other predator sounds, masked or disrupted odontocete returning echolocation [21, 24, 33–36];
- Passive acoustic decoys and deterrents such as the incorporation of objects into gear that: simulate the acoustic target strength of odontocete-depredated catch, simulate the target strength of species that odontocetes avoid depredating, are perceived as unusual prey, or interfere with echolocation [24, 37];
- Acoustic masking, including quieter vessels; bubble screen to reduce the dissemination of vessel sounds; broadcast of sounds to conceal the sounds of the vessel gear, setting and hauling; not remaining near the gear after it is set; and minimizing shifting in and out of gear [20];
- Artificial bait [38];
- Weak hooks. While there is no evidence of weak hooks causing reduced odontocete bycatch rates, mechanistic studies found that wire diameter was an informative predictor of the force required to straighten hooks [39–41] and during at-sea experiments weak hooks were observed to straightened more often than control hooks with a wider hook wire diameter [39, 40]; and
- Chemical deterrents [20, 24, 42].

There is also weak evidence of the effect of prescribed pelagic longline handling and release practices on cetacean post-release mortality rates. For instance, estimates of survival probability made by the US Government are based largely on expert opinion [22, 43]. Therefore, evidence is needed to support the identification and development of effective and commercially viable approaches to mitigate odontocete depredation and bycatch rates in pelagic longline fisheries.

Understanding the depredation strategies of odontocetes that are susceptible to fisheries bycatch can contribute to mitigating this human-wildlife conflict. Knowledge of species-specific odontocete depredation rates might contribute to the development of promising, new mitigation methods, such as geospatial, depth and temporal separation of predictable catch rate hotspots of species with both low commercial value and high odontocete depredation rates and hotspots of catch rates of principal market species, and passive acoustic decoys, deterrents and echolocation disruptors. This study analyzed observer data from the Hawaii tuna longline fishery to estimate teleost and elasmobranch species-specific mean posterior marine mammal depredation rates using a Bayesian binomial likelihood estimator with a Bayes-Laplace prior, expanding upon previous assessments [44, 45]. The study also assessed the correlation between species-specific depredation rates and length. Study findings provide evidence to support the innovation of effective and commercially viable methods to reduce the risk of odontocete depredation and bycatch in pelagic longline fisheries.

## 2. Methods

Data on species-specific catch, presence/absence of marine mammal depredation and length were obtained from the U.S. government observer program database for the Hawaii pelagic longline fishery. Observer data fields and data collection protocols are defined in NMFS [46]. The study period was from 15 August 2003, the date when observers began to record damage to the catch, through 26 August 2023, the most recent available record. The study sample included 179,853,812 hooks deployed in 73,217 sets within 5,445 trips by 194 vessels. Records were included in the study sample to estimate species-specific odontocete depredation rates for fish species, both teleosts and elasmobranchs, that were identified to the species level, and had >100 capture records where the observer determined whether the catch had been depredated by a marine mammal, Thus, the study sample excludes catch records where either the observer was unable to determine whether there was damage to the catch or observed damage but could not determine the source. Length measurement methods of eye-to-fork was used for billfishes and fork length for all other species, as these produced the largest sample sizes for species within these groups.

The expected depredation rate (or proportion) for each of the 50 species was estimated using a Bayesian binomial likelihood estimator that here accounts for the zero recorded depredation events [47]. A simple binomial estimator is the most appropriate likelihood for these data that comprise the number of depredated observations and the number of not-depredated observations per set (or “successes” and “failures”)—see Lin and Chu [48] for further discussion. Moreover, 40% of the 35 species-specific records had zero depredations per set and hence the use of a Bayes-Laplace prior to account for those zero observations explicitly when using a binomial estimator ([47]; and see Gilman et al. [49] for an example for shark at-vessel mortality rates). The mean posterior rate and a 95% highest posterior density interval (HDI) was summarized by sampling from a binomial likelihood with a Bayes-Laplace prior [47] using the binom package for R [50] as proposed elsewhere by Roda et al. [51] and Gilman et al. [49]—rather than just using the raw study-specific summaries.

The relationship between predicted mean depredation rates and median lengths was assessed for teleosts with > 1,000 capture records by calculating the Pearson product-moment correlation coefficient [52] using the corr() and cor.mtest () functions in the corrplot R package [53]. There were insufficient sample sizes available to estimate expected marine mammal depredation rates by length class within species (of 1,038,586 species-level catch records with length values, only 44 were identified as having marine mammal depredation)–observers are typically unable to make common length measurements (total length, eye-to-fork length, fork length) of catch depredated by marine mammals because the depredated catch tends to be retrieved with only the fish head up to the gills, or just the lips and upper jaw of the fish remaining [15, 16]. In other words, we were able to conduct analyses to determine whether catch with larger median lengths had different expected mean odontocete depredation rates than species with smaller median lengths, but not on whether larger individuals within a species had a different odontocete depredation rate than smaller individuals of that species.

The proportion of total sets with odontocete depredation of ≥1 fish was determined. The proportion of the total catch of bigeye tunas (*Thunnus obesus*), yellowfin tunas (*T*. *albacares*) and combined teleosts that had odontocete depredation was determined for (1) sets with observed odontocete depredation of ≥ 1 fish, and (2) sets with observed odontocete depredation of ≥ 1 bigeye tuna, yellowfin tuna or teleost. We then also compared the depredation rate for bigeye and yellowfin tunas, the principal target species of this fishery, using the odds ratio [54] calculated by conditional maximum likelihood or Fishers exact procedure using the epitools R package [55].

## 3. Results

Table 1 presents the mean posterior depredation rates (percent of the catch that was depredated by marine mammals) with 95% HDIs and median and mean lengths. For species with >1,000 capture records, depredation rates ranged from a high of ca. 1.2% (1.1 to 1.3 95% HDI) for shortbill spearfish to a low of 0.002% (0.001 to 0.003 95% HDI) for blue shark. Bigeye tuna was at least two times more likely to be depredated than yellowfin tuna (odds ratio = 2.03, 95% CI: 1.8–2.3, P<0.0001). Elasmobranchs had extremely low marine mammal depredation rates, where of a total of 442,377 captured elasmobranchs identified to the species level and for which the observer determined whether marine mammal depredation occurred, 7 were depredated (2 bigeye thresher sharks and 5 blue sharks) (Table 1).

**Table 1:** Study sample sizes, mean posterior depredation rates and 95% highest posterior density intervals (HDIs), and length summaries for fish species with >100 catch records for which an observer determined whether there was marine mammal depredation in the Hawaii longline tuna fishery (2003–2023). There were 179,853,812 observed hooks in 73,217 sets. Lengths are eye-to-fork for billfishes and fork length for all other species. Sorted from high to low mean depredation rate within 5 taxonomic groups.

Scientific name	Common name	% depredated			No. caught	No. depredated	Length (cm)		
		mean	lower HDI	upper HDI			N	Median	Mean
**Tunas**									
*Katsuwonus pelamis*	Skipjack tuna	0.469	0.433	0.505	136,931	641	51,940	71	70
*Thunnus obesus*	Bigeye tuna	0.292	0.280	0.305	735,284	2,148	288,006	113	112
*Thunnus alalunga*	Albacore tuna	0.167	0.134	0.202	55,066	91	22,272	101	100
*Thunnus albacares*	Yellowfin tuna	0.144	0.127	0.162	177,441	255	68,398	117	110
**Billfishes**									
*Tetrapturus angustirostris*	Shortbill spearfish	1.172	1.088	1.256	62,722	734	24,320	134	134
*Xiphias gladius*	Swordfish	0.466	0.389	0.545	29,386	136	10,643	93	109
*Kajikia audax*	Striped marlin	0.387	0.338	0.436	61,259	236	26,001	137	134
*Istiophorus platypterus*	Indo-Pacific sailfish	0.263	0.077	0.475	2,283	5	800	145	141
*Makaira nigricans*	Blue marlin	0.229	0.165	0.297	20,055	45	7,646	161	166
**Other teleosts**									
*Elagatis bipinnulata*	Rainbow runner	1.775	0.185	3.764	167	2	34	73	75
*Acanthocybium solandri*	Wahoo	1.060	0.992	1.128	87,450	926	24,876	127	126
*Uraspis secunda*	Cottonmouth jack	0.952	0	2.839	103	0	28	32	31
*Brama japonica*	Pacific pomfret	0.877	0	2.616	112	0	74	33	35
*Lampris megalopsis*	Bigeye Pacific opah	0.579	0.524	0.635	71,311	412	27,415	101	100
*Assurger anzac*	Razorback scabbardfish	0.386	0	1.154	257	0	77	224	218
*Coryphaena hippurus*	Dolphinfish	0.332	0.310	0.353	278,395	922	77,893	85	86
*Masturus lanceolatus*	Sharptail mola	0.331	0	0.990	300	0	16	94	93
*Trachipterus fukuzakii*	Tapertail ribbonfish	0.253	0.005	0.601	790	1	187	194	190
*Nesiarchus nasutus*	Black gemfish	0.250	0	0.748	398	0	144	70	67
*Ranzania laevis*	Slender mola	0.218	0.099	0.349	5,037	10	1,196	50	51
*Mola mola*	Common mola	0.211	0	0.630	473	0	4	98	113
*Promethichthys prometheus*	Roudi’s escolar	0.112	0	0.336	889	0	253	64	65
*Sphyraena barracuda*	Great barracuda	0.105	0.045	0.172	9,488	9	2,762	99	101
*Scombrolabrax heterolepis*	Longfin escolar	0.105	0.039	0.179	7,595	7	1,889	24	24
*Omosudis lowii*	Hammerjaw	0.103	0	0.309	968	0	236	27	28
*Eumegistus illustris*	Lustrous pomfret	0.095	0	0.284	1,054	0	439	49	51
*Taractichthys steindachneri*	Sickle pomfret	0.085	0.074	0.097	230,917	196	96,573	60	58
*Lepidocybium flavobrunneum*	Escolar	0.079	0.067	0.093	179,932	142	47,235	76	76
*Lagocephalus lagocephalus*	Pelagic puffer	0.066	0	0.198	1,510	0	369	46	45
*Gempylus serpens*	Snake mackerel	0.062	0.052	0.071	256,820	157	63,820	99	101
*Taractes asper*	Rough pomfret	0.055	0	0.165	1,815	0	611	50	53
*Coryphaena equiselis*	Pompano dolphinfish	0.049	0	0.146	2,051	0	666	57	58
*Alepisaurus ferox*	Longnose lancetfish	0.032	0.028	0.035	855,920	270	179,579	113	105
*Ruvettus pretiosus*	Oilfish	0.032	0.001	0.076	6,228	1	1,686	72	86
*Taractes rubescens*	Dagger pomfret	0.012	0	0.028	17,135	1	5,676	67	64
**Rays**									
*Mobula mobular*	Devil ray	0.261	0	0.781	381	0	0	NA	NA
*Pteroplatytrygon violacea*	Pelagic stingray	0.004	0	0.012	24,375	0	27	52	59
**Sharks**									
*Carcharhinus galapagensis*	Galapagos shark	0.926	0	2.761	106	0	1	115	115
*Carcharhinus plumbeus*	Sandbar shark	0.595	0	1.778	166	0	5	109	121
*Sphyrna zygaena*	Smooth hammerhead shark	0.301	0	0.901	330	0	13	197	191
*Isurus paucus*	Longfin mako shark	0.112	0	0.334	894	0	95	156	158
*Alopias pelagicus*	Pelagic thresher shark	0.099	0	0.297	1,006	0	87	125	122
*Zameus squamulosus*	Velvet dogfish	0.020	0	0.059	5,106	0	1,013	69	68
*Carcharhinus falciformis*	Silky shark	0.019	0	0.058	5,153	0	270	108	114
*Pseudocarcharias kamoharai*	Crocodile shark	0.018	0	0.053	5,618	0	642	85	84
*Carcharhinus longimanus*	Oceanic whitetip shark	0.015	0	0.046	6,475	0	363	106	107
*Alopias superciliosus*	Bigeye thresher shark	0.009	0.001	0.018	34,728	2	596	156	157
*Isurus oxyrinchus*	Shortfin mako shark	0.006	0	0.018	17,072	0	1,012	185	186
*Prionace glauca*	Blue shark	0.002	0.001	0.003	340,967	5	566	175	176

**Fig 1:**
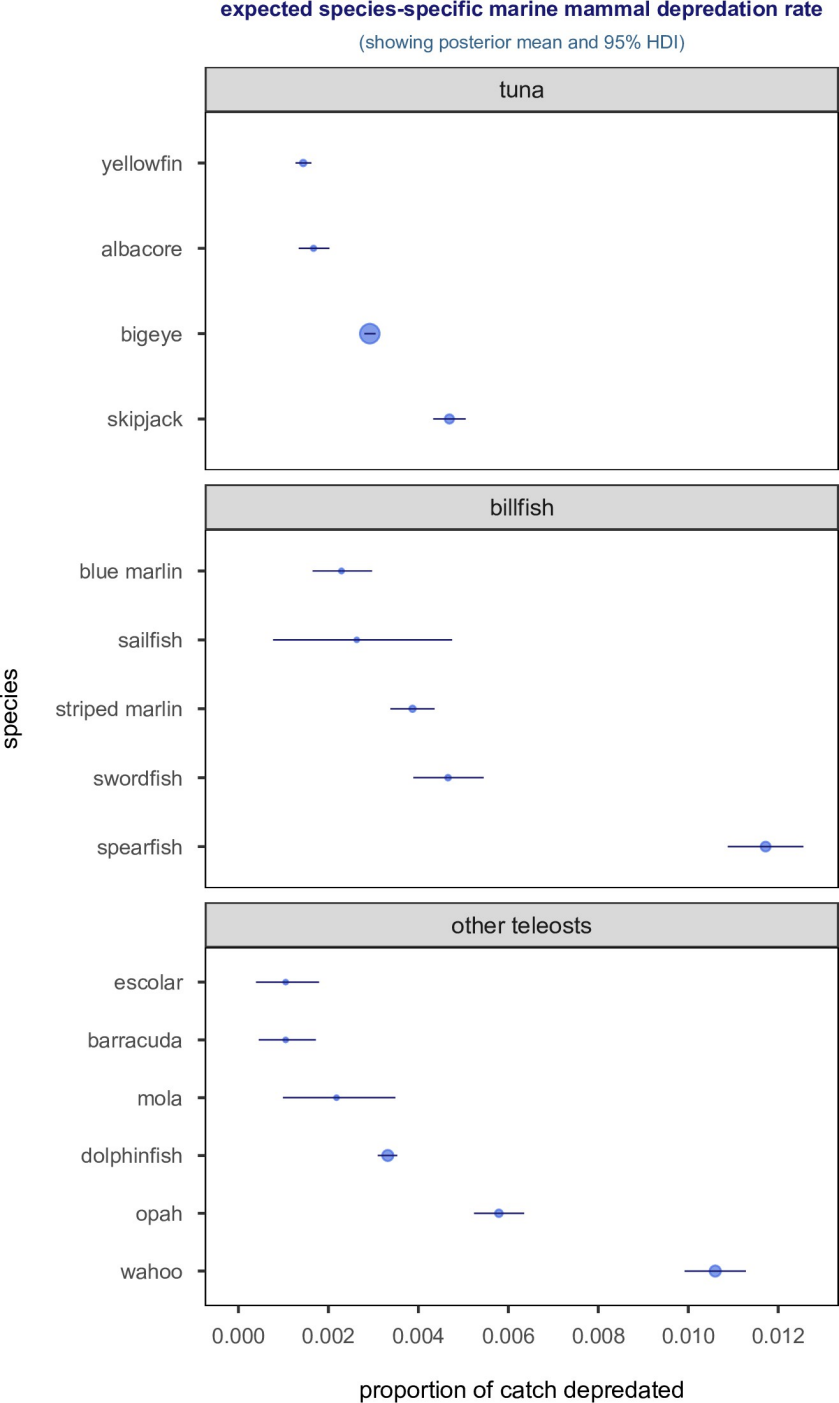
Mean posterior depredation rates (proportion of the number captured that was depredated by odontocetes) and 95% highest posterior density intervals (HDIs) for tunas, billfishes and six other teleost species with the highest predicted mean depredation rates and with >1,000 capture records in the Hawaii tuna longline fishery (2003–2023). Dot size is proportional to catch for that species relative to the catch for all three taxonomic groupings.

Fig 1 presents the predicted mean depredation rate (proportion of the catch with marine mammal depredation) and 95% HDIs for the four species of tunas included in Table 1, the five species of billfishes included in Table 1, and the six species of other teleosts with the highest predicted mean depredation rates and with sufficient sample sizes of >1,000 capture records. Species are sorted from lowest to highest depredation rate within each of the three taxonomic groups.

Consistent with this species-selective depredation, there was high variability in the species-specific set-level marine mammal depredation rates. For example, of sets with ≥ 1 depredated bigeye tuna, the main target species of this fishery, 94% had depredation of over half of the captured bigeye tuna. This rate was 42% for yellowfin tuna (i.e., of sets with ≥ 1 depredated yellowfin tuna, 42% had depredation of over half of the captured yellowfin tuna), and 2% for combined teleost species. Similarly, for sets with depredation of ≥ 1 fish of any species, 10%, 4% and 2% of these sets had depredation of over half of the captured bigeye tuna, yellowfin tuna and combined teleosts, respectively. For sets with depredation of ≥ 1 fish of any species, 4%, 2% and 0.1% of these sets had odontocete depredation of all the captured bigeye tuna, yellowfin tuna and combined teleosts, respectively. Of the sets in the study sample, 6.2% had observed odontocete depredation of ≥ 1 fish.

There was no significant relationship between median length and mean depredation rate: Pearson’s r = 0.14 (95% CI: -0.26 to 0.50), p = 0.49. The species-specific median length of the catch was not an informative predictor of odontocete depredation rate of teleosts.

## 4. Discussion and conclusions

Marine mammal depredation is a rare event in this fishery, occurring in about 6% of sets. When depredation did occur, most frequently a small proportion of the total catch was depredated. However, there was large variability in odontocete depredation rates between teleost species, while all species of captured elasmobranchs had negligible odontocete depredation. This is consistent with previous studies of the observer program database for the Hawaii tuna longline fishery [44, 45]. Findings suggest that odontocetes selectively depredate a subset of the teleost species captured within sets. Odontocetes are not indiscriminately depredating the catch down the line, but are selectively depredating certain teleost species.

Different species of pelagic marine predators, and sizes within species, have different prey preferences and prey species-specific predation behavior [38], as suggested by the species-selective depredation observed in this study. For some odontocetes, predation and other behaviors may also vary by sympatric populations (e.g., for killer whales in the northeast Pacific Ocean, transients feed on marine mammals and residents target fishes, [56]). Pelagic marine predators’ detection, search and attack behavior and preferences for different prey species might be a function of prey species’ net energy value (i.e., the prey’s caloric value compared to the energy required to catch and handle the prey); and chemical, visual (e.g., size, shape, color and movement), acoustic and textural characteristics [38, 57–62]. However, median length was found in this study to be unrelated to depredation rate. Globally, false killer whales have a broad diet that includes coastal and demersal fishes and epipelagic and oceanic-neritic squids, as well as pelagic fishes that are also captured in pelagic longline fisheries, including commercial species such as tunas, dolphinfish, wahoo and sickle pomfret (monchong) [44, 63, 64].

Observers often cannot determine the species of odontocete depredated catch from the small portion of the carcass remaining on the gear. The species-specific odontocete depredation rates for teleosts are conservative underestimates because they do not account for over half of records of odontocete depredated teleosts for which the observer could not identify the catch to the species level. A relatively small proportion of total captured tunas, billfishes and other teleosts lacking odontocete depredation were not identified to the species level. Observers recorded all seven odontocete depredated sharks (no rays were odontocete depredated) to the species level while some captured elasmobranchs without odontocete depredation were not identified to the species level, and therefore the elasmobranch species-specific depredation rates may be conservative overestimates. The observer program database also lacks records of pre-catch losses, including when odontocetes and other predators completely remove catch from the gear prior to gear retrieval, as well as catch that falls from the gear due to mechanical action, representing an additional data limitation that affects the accuracy of predicted species-specific odontocete depredation rates.

More robust estimates of species-specific odontocete depredation rates could be derived through research using observer data from longline fisheries that standardize fishing effort to account for potentially informative predictors of odontocete depredation. As conduced previously to explore a response of odontocete depredation of combined species of catch, models with a response of species-specific depredation rate could include predictors such as unique vessel, consecutive sets within a trip, season, geospatial location, hooks per set, mainline length, soak duration, bathymetry and sea surface temperature [26, 32, 37, 45].

While a rare event, odontocete depredation may cause substantial economic and operational costs in the Hawaii tuna longline fishery. This includes direct costs from lost catch [16, 65]. For example, Fader et al. [16] estimated that in the Hawaii tuna longline fishery, odontocete depredation of bigeye and yellowfin tunas and dolphinfish, the three most frequently retained species in this fishery, had an annual value of USD 1 million. Other economic costs result from odontocete depredation of bait, lost and damaged fishing gear and time for crew to repair and replace it, lost fishing time when vessels move or wait to make another set after observing depredation, and increased fishing effort to make up for lost catch [15, 16].

Depredation also leads to bycatch fishing mortality and sublethal effects such as altered foraging behavior, distributions, diet and demographics [7, 17–19]. The false killer whale is the main odontocete species captured in the Hawaii fishery. During the study period (2003–2023) about 70% of captured marine mammals were false killer whales (of 173 odontocetes identified to the species level or higher taxonomic group that could be differentiated from false killer whales). The main Hawaiian Islands insular false killer whale population segment is listed as endangered under the U.S. Endangered Species Act [66], while trends in the abundance of other false killer whale population segments that are exposed to the Hawaii longline and other fisheries are highly uncertain [67].

There are no known odontocete depredation and bycatch mitigation methods for pelagic longline fisheries across the tiers of a sequential mitigation hierarchy that are effective, do not harm odontocetes, and are likely to have acceptable costs on economic viability, practicality and crew safety [15, 21, 24, 25, 45]. To date, measures recommended by an advisory team formed under the U.S. Marine Mammal Protection Act and interventions adopted by the national management authority (weak circle hooks, permanent static closed area, fleetwide bycatch cap that triggers an area closure, and prescribed handling and release practices) have been unsuccessful in meeting management objectives [22].

Study findings might provide evidence to support the identification and innovation of effective and commercially viable methods to mitigate odontocete fisheries depredation and bycatch risk. For example, the spatial and temporal distribution of catch rates of species with and without relatively high odontocete depredation rates could be explored. Fishing effort could be conducted in areas and periods with relatively low catch rates of species with low commercial value and with high odontocete depredation rates that maintains economic viability but lowers odontocete depredation and catch rates [68, 69]. Managing the fishing depth and time of day of fishing might similarly enable reducing catch rates of species with high odontocete depredation rates that also have low commercial value [16, 70]. These findings on odontocete depredation strategies might also contribute to the development of passive acoustic decoys and deterrents. Incorporating objects into the fishing gear may act as decoys if they effectively simulate the acoustic target strength (TS) of the main depredated catch species–hence confusing or frustrating depredating whales [24, 37]. Or, if the objects mimic the TS of a species that odontocetes avoid depredating, then the object may effectively deter depredation behavior [37], such as sharks, which lack swimbladders and have a low TS relative to teleosts. For species with swimbladders, the swimbladder accounts for most (>90%) of fishes’ acoustic backscatter [71, 72]. Similarly, hollow alloy spheres attached near terminal tackle have been proposed to reduce odontocete-longline depredation where the object might cause odontocetes to perceive the prey as unusual or the sphere might interfere with their echolocation [73]. This expanded evidence of the species-selective depredation strategies of odontocetes that are exposed to fisheries bycatch promises to contribute to mitigating this human-wildlife conflict.
